# VEGF Receptor 1 Promotes Hypoxia-Induced Hematopoietic Progenitor Proliferation and Differentiation

**DOI:** 10.3389/fimmu.2022.882484

**Published:** 2022-05-12

**Authors:** Jonathan Florentin, Scott P. O’Neil, Lee L. Ohayon, Afaz Uddin, Sathish Babu Vasamsetti, Anagha Arunkumar, Samit Ghosh, Jennifer C. Boatz, Justin Sui, Corrine R. Kliment, Stephen Y. Chan, Partha Dutta

**Affiliations:** ^1^Division of Cardiology, Department of Medicine, Pittsburgh Heart, Lung, Blood, and Vascular Medicine Institute, University of Pittsburgh Medical Center, University of Pittsburgh School of Medicine, Pittsburgh, PA, United States; ^2^Department of Medicine, Division of Pulmonary and Critical Care, University of Pittsburgh, Pittsburgh, PA, United States; ^3^Department of Immunology, University of Pittsburgh School of Medicine, Pittsburgh, PA, United States

**Keywords:** VEGFR1, hematopoietic progenitor, intermittent hypoxia, inflammation, innate immune cell

## Abstract

Although it is well known that hypoxia incites unleashed cellular inflammation, the mechanisms of exaggerated cellular inflammation in hypoxic conditions are not known. We observed augmented proliferation of hematopoietic stem and progenitor cells (HSPC), precursors of inflammatory leukocytes, in mice under hypoxia. Consistently, a transcriptomic analysis of human HSPC exposed to hypoxic conditions revealed elevated expression of genes involved in progenitor proliferation and differentiation. Additionally, bone marrow cells in mice expressed high amount of vascular endothelial growth factor (VEGF), and HSPC elevated VEGF receptor 1 (VEGFr1) and its target genes in hypoxic conditions. In line with this, VEGFr1 blockade *in vivo* and *in vitro* decreased HSPC proliferation and attenuated inflammation. *In silico* and ChIP experiments demonstrated that HIF-1α binds to the promoter region of *VEGFR1*. Correspondingly, *HIF1a* silencing decreased VEGFr1 expression in HSPC and diminished their proliferation. These results indicate that VEGF signaling in HSPC is an important mediator of their proliferation and differentiation in hypoxia-induced inflammation and represents a potential therapeutic target to prevent aberrant inflammation in hypoxia-associated diseases.

## Introduction

Diseases characterized by alveolar hypoxia, such as chronic obstructive pulmonary disease (COPD) and obstructive sleep apnea (OSA), are highly prevalent and contribute to significant morbidity and mortality around the world (1–5). Hypoxia-associated diseases bear high comorbidities including metabolic, cardiovascular, neoplastic and neurologic disease, which a growing body of literature attributes to systemic inflammation (1, 6–8). To date, studies of alveolar hypoxia induced inflammation have focused primarily on the role of resident macrophages, and their interaction with alveolar endothelial cells mediating production of inflammatory cytokines (9–12). Monocyte chemoattractant protein 1 (MCP-1), c-reactive protein, tumor necrosis factor alpha (TNFα), IL-1, IL-6 and IL-8 are increased in hypoxic lung diseases and generally correlate with severity of disease (11, 13, 14). While there is strong evidence that local and systemic inflammation are key components of hypoxic diseases, the mechanisms of exaggerated cellular inflammation in hypoxic conditions have yet to be defined.

Mounting evidence has demonstrated key inflammatory cells involved in the pathogenesis of various diseases including insulin resistance and atherosclerosis (4, 15–17). These cells have been proposed to originate from hematopoietic stem and progenitor cells (HSPC) in the bone marrow and spleen (18–21). Patients with hypoxic lung disease exhibit elevated inflammation and leukocytosis. The leukocytosis observed in chronic inflammation likely represents production of inflammatory leukocytes by their progenitors. However, a direct link between hypoxia induced systemic inflammation and proliferation of HSPC has not been established. Furthermore, the mechanisms of HSPC proliferation and differentiation in hypoxia have not been studied.

Vascular endothelial growth factor (VEGF) has been identified as an important regulator of neovascularization by encouraging endothelial cell proliferation in various diseases such as age-related macular degeneration and neoplastic diseases (22–25). Several recent studies have demonstrated elevated VEGF concentrations in peripheral blood of patients with hypoxic lung disease, which closely correlated with severity of disease (26–31). However, the role of VEGF in HSC proliferation and inflammatory leukocyte production is not known.

We used two independent mouse models of hypoxia to understand the effect of hypoxia in HSPC proliferation: C57BL/6 mice exposed to A) 10% O_2_ for three weeks, an established mouse model of chronic hypoxia (32–34) and B) cigarette smoke for six months, a COPD mouse model (35, 36). Mice under chronic hypoxic conditions and exposed to cigarette smoke had elevated numbers of neutrophils, monocytes and macrophages in the blood and lungs suggesting augmented hematopoiesis. In line with this, a whole genome RNA sequencing analysis of human HSPC cultured under hypoxic conditions showed increased expression of the genes involved in hematopoiesis. The number and proliferation of HSPC were increased when mice were exposed to hypoxia or cigarette smoke. Concomitantly, we measured increased expression of VEGFr1 in these activated progenitors indicating a possible role of VEGFr1 in hematopoiesis. Indeed, VEGFr1 blockade *in vitro* and *in vivo* diminished HSPC proliferation and their differentiation into myeloid cells, resulting in attenuated inflammation in hypoxic conditions. Mechanistically, we observed that HIF-1α binds to the *VEGFr1* promoter and increases its expression in HSPC under hypoxic conditions. *HIF1a* silencing reduced HSPC proliferation and their differentiation into inflammatory leukocytes.

As a whole, the present study describes a role of the VEGFr1 in the activation of hematopoietic progenitors and the production of inflammatory myeloid cells in hypoxia. Additionally, this work proposes the VEGFr1 as a possible therapeutic target to attenuate inflammatory burden in patients with hypoxia.

## Methods

### Animals

All animal experiments were conducted following NIH and ARRIVE (Animal Research: Reporting of *In Vivo* Experiments, https://arriveguidelines.org) guidelines under protocols approved by the Institutional Animal Care and Use Committee of the University of Pittsburgh. Ten to twelve-week-old C57BL/6J male mice were exposed to normobaric hypoxic–10% oxygen, which represents 50% of the normal amount of oxygen, or normoxic conditions for 3 weeks. This oxygen concentration resulted in increased right ventricular systolic pressure, Fulton index and pulmonary vascular remodeling (37, 38). Of note, we have observed a very high mortality when we further decreased O_2_ concentration. After 21 days in hypoxic chamber, mice underwent right ventricular catheterization, followed by tissue and blood collection. The mice were anesthetized with ketamine/xylazine and ventilated through a transtracheal catheter.

### Flow Cytometry

C57BL/6 mice were anesthetized, and a small volume of peripheral blood was collected by cardiac puncture, followed by transcardial perfusion with 15mL of ice-cold PBS. One lobe of the lung was harvested, minced and digested with collagenase I, collagenase XI, and hyaluronidase for one hour at 37C. After incubation, a single cell suspension was prepared by passing the digested lung tissue through a 70 μm nylon strainer, followed by washing with 0.5% bovine serum albumin in PBS (FACS buffer). The filtrate was washed with 10 mL of FACS buffer and centrifuged at 4°C for 7 minutes at 350 g. The supernatant was discarded, and the samples were re-suspended and labeled with 600-fold diluted antibody mixture with the following antibodies: anti-CD45.2 (BD Biosciences clone 104, cat#560693), CD11b (BD Biosciences, clone M1/70, cat#557657), CD115 (BD Biosciences, clone T38-320, cat# 565249), Ly6G (BD Biosciences, clone 1A8, cat#564979) and CD64 (BD Biosciences, clone X54-5/7.1, cat#558455). B and T cells were identified as CD45+, CD11b-, and CD19+ or CD3+, respectively. Monocytes were identified as CD45+, CD11b+, CDLy-6G- and CD115+. Neutrophils were considered as CD45+, CD11b+, CD115- and Ly6G+. For bone marrow hematopoietic stem and progenitor cells analyses, cells were stained with biotin conjugated antibodies against lineage markers including B220 (Biolegend, clone RA3-6B2, cat# 103203), CD4 (GK1.5, BD Biosciences, cat# 555345), CD8a (53-6.7, BD Biosciences, cat# 555365), NK1.1 (PK136, Biolegend, cat# 109704), CD11b (M1/70, Biolegend, cat# 101204), CD11c (N418, Biolegend, cat# 117304), Gr-1 (Biolegend, clone RB6-8C5, cat# 108403) and, Ter119 (TER-119, Biolegend, cat# 116204) followed by streptavidin APC/Cy7 conjugation, and antibodies against c- Kit (2B8, Biolegend, cat# 105822), Sca-1 (D7, Biolegend, cat# 108120), IL7Ra (SB/199, BDBiosciences, cat# 565490), CD16/32 (2.4G2, Biolegend, cat# 101318), CD34 (RAM34, BD Biosciences, cat# 553733), CD48 (HM48-1, Biolegend, cat# 103418) and CD150 (TC15-12F12.2, Biolegend, 115918). Hematopoietic stem cells (HSC) were identified as Lin- c-Kit+ Sca-1+ CD48- CD150+, and LSK were deemed to be Lin- c-Kit+ Sca-1+. Granulocyte-macrophage progenitors (GMP) were identified as Lin- c-Kit+ Sca-1- CD16/32+ CD34+. Samples were incubated on ice for 1 hour. Cell numbers per femur were calculated by multiplying total cell counts using a hemocytometer by cell subset frequencies obtained from flow cytometry analysis. Murine Vegfr1 expression was assessed using a Vegfr1 antibody (Thermofisher Scientific, clone 3A6, cat # BSM-52338R). A Fortessa Flow Cytometer (BD) was used to acquire data. Data were analyzed with FlowJo software (Tree Star).

### Biochemical Assays

Concentrations of cytokines in plasma and parenchymal tissues were quantified by enzyme-linked immunosorbent assay (ELISA). IL-6 levels were quantified in lung tissue from hypoxic and normoxic conditions using a mouse IL-6 capture ELISA kit following the manufacturer instructions (Invitrogen, Cat # BMS603-2). Comparisons between conditions were performed using Graphpad Prism, and absolute concentration was calculated based on a lyophilized standard included in the kit.

### RT PCR

Lungs were harvested and immediately snap frozen in liquid nitrogen until they could be processed. RNA extraction was performed using the PicoPure RNA isolation kit (Applied BioSystems, Cat # KIT0204) without modification to the manufacturer protocol. Total RNA was quantified using a NanoDrop spectrophotometer. Complementary DNA was generated from 100 ng of mRNA per sample using the high capacity cDNA Reverse Transcription kit (Applied BioSystems, Cat # 4368814). Relative gene expression was determined by qPCR with the PowerUp SYBR Green reporter (Applied BioSystems, Cat # A25742) and primers supplied by IDT; gene expression was represented as ΔCt normalized to *beta-actin* expression.

### Whole Mount Imaging

Freshly dissected sternums from C57BL/6 mice exposed to normoxic or hypoxic conditions were fixed in cold 4% paraformaldehyde (PFA, ThermoFisher Scientific, Cat # 28908) in PBS for 25 minutes without agitation. The bones were washed in PBS three times with 15-minute incubations before incubating with common lineage biotinylated antibodies, including anti-TER119 (Biolegend, clone TER-119, cat#116204), CD11b (BD Biosciences, clone M1/70, cat#553309), CD11c (Biolegend, clone N418, cat#117304), CD45R/B220 (Biolegend, clone RA3-6B2, cat#103204), NK1.1 (Biolegend, clone PK136, cat#108704), GR-1 (Biolegend, clone RB6-8C5, cat#108404), CD4 (Biolegend, clone GK1.5, cat#100404), CD8 (Biolegend, clone 53-6.7, cat#100704) and CD127 (Biolegend, clone A7R34, cat#135006). Samples were incubated for 24 hours at 4° C, then washed three times with 1% FBS in PBS with 15-minute incubations, then labeled with streptavidin FITC antibody, followed by overnight incubation at 4°C. The samples were sequentially labeled with anti-VE Cadherin (BD Biosciences, clone 11D4.1, cat# 562242) and anti-CD31 APC antibodies (BD Biosciences, clone MEC 13.3, cat# 551262), followed by rabbit anti-mouse CD150 then rat anti-rabbit Cy3 (Invitrogen) with three wash steps between primary and secondary antibody overnight incubations. Lastly, the samples were washed three times and imaged on a Nikon A1 confocal microscope; Images were analyzed using ImageJ software.

### Human Whole Blood data

All human data were collected in accordance with a University of Pittsburgh Independent Review Board approved protocol. Patients with intermittent hypoxia, defined by an apnea hypoxia index (AHI) > 5, were eligible for inclusion in the obstructive sleep apnea group, and matched controls with AHI less than 5 were eligible for inclusion in the control group. Patients with pulmonary function testing (PFT) confirmed chronic obstructive lung disease were eligible for inclusion in the COPD group. Patients with concurrent conditions known to cause leukocytosis were excluded, including known history of CVD, dyslipidemia, diabetes mellitus, allergic, pulmonary or hematological disease, malignancy, recent injury or surgery, recent systemic steroid use, current infection or any systemic medication use (*e.g.* hypolipidemic or anti-platelet agents). Complete blood cell count laboratory data were collected prior to medical interventions.

### HSPC Cell Culture

Hematopoietic stem and progenitor cells were enriched by negative magnetic-activated cell sorting from vertebral, femoral and tibial bone marrow (StemCell Technologies, Cat # 17665). Briefly, we crushed the bones and filtered the cells through a 40μm mesh filter. The cells were then spun down and resuspended in 1mL of FACS buffer. They were then stained with biotin-conjugated lineage antibodies (B220, TER119, Ly6G, CD4, CD8, CD11b, CD11c, and IL7R) at 1:300 dilution for 20 minutes on ice. The cells were spun down and resuspended in 2 ml of buffer (DPBS+2%FBS+1mM EDTA), and 100 ul of biotin selection cocktail was added for 15 minutes at room temperature. Another 50 μL of magnetic nanoparticles was added to the mix for 10 minutes at room temperature. The cells were then placed in a FACS tube in an EasySep magnet (Stem Cell Technologies, Cat# 18000). After 6-7 minutes, the unbound cells were collected. Cells were then cultured in the presence of G-CSF on 96 well round-bottom cell culture plates with 10, 50, or 100 nM concentrations of Sugen (SU5416, MedChem Express), or with siRNA control (si*Ctl*, IDT) or against *Vegfr1* (si*Vegfr1*, IDT), for 72 hours prior to hypoxia challenge. Samples were exposed to normobaric hypoxic conditions (10% O_2_) for 24 hours prior to RNA extraction and flow cytometry analysis.

### RNA Sequencing

Publicly available differentially expressed genes from HSPC exposed to hypoxic conditions (https://www.ncbi.nlm.nih.gov/geo/query/acc.cgi?acc=GSE54663) were assessed for genes of interest including VEFGA and VEGFr1, and heatmap representations of the data were prepared. Enriched pathways were discerned using Ingenuity Pathway Analysis.

### ChIP Sequencing

To assess the interaction between HIF-1a and VEGFr1, we first located the promoter region of VEGFr1 using the UCSC genome browser (University of California Santa Cruz). Then, we looked for potential HIF-1-alpha binding sites onto the promoter region of VEGFr1 using Transfac software (GeneXplain). The primers for each HIF-1α binding site were designed using NCBI Primer-BLAST. Murine HSPC were harvested from the bone marrow of C57BL/6 mice. Cells were resuspended in complete SFEM media and plated onto 10 cm dishes. Cells were incubated for 24 hours in normoxia or hypoxia. Cells were then harvested and resuspended in PBS at a concentration 10^6^ cells/mL. Cells were then fixed, and DNA was extracted and immunoprecipitated by a ChIP grade HIF-1α antibody (Rb polyclonal, Novusbio, Cat # NB100-479SS) as previously described (39). Finally, real time PCR was run to quantify the amount of *HIF1A* bound to *VEGFr1* promoter region in each condition.

### Adoptive Transfer Experiment

C57BL/6 mice were placed under either normoxic or hypoxic conditions (10% O_2_) for 10 days. At day 7 of hypoxia, bone marrow HSPC from KIkGR^+^ (Kikume Green Red) mice were negatively enriched using a magnetic separation method and retro-orbitally injected into either normoxic or hypoxic C57BL/6 mice at a concentration of 20 million cell/mL. Mice were sacrificed, and lungs, bone marrow, spleen and blood were harvested at day 10 of hypoxia. The percentages of progenies derived from the adoptively transferred cells were measured by flow cytometry.

### Statistical Analysis

Data were compiled using Prism (GraphPad). Statistics were generated and are presented as the mean ± SEM. Statistical significance between two categories of analyzed samples was calculated using two-tailed Student’s *t* tests. For multiple category comparisons, one-way ANOVA was used with a *post hoc* Bonferroni test. Differences with *P* values <0.05 were considered statistically significant.

## Results

### Hypoxic Mice Have Elevated Numbers of Inflammatory Leukocytes in the Blood

Various diseases associated with intermittent or chronic hypoxia are characterized by exaggerated systemic inflammation (5, 6, 16, 17, 40–43). Leukocytes, primarily myeloid cells, play a major role in inflammatory diseases including atherosclerosis and diabetes (15, 42). To study alteration in hematopoietic progenitors and their lineage output, we used two established mouse models of hypoxia- mice housed in hypoxic chambers containing 10% oxygen for three weeks and mice exposed to cigarette smoke for six months (35, 36). Mice exposed to cigarette smoke contained significantly heightened numbers of lymphocytes, such as B and T cells, in the bone marrow while myeloid cell content decreased (**Supplementary Figures 1A, B**). In contrast, splenic lymphocyte numbers were unaltered (**Supplementary Figure 1C**), and myeloid cells were more numerous in smoke-exposed mice compared to the control group (**Supplementary Figure 1C**). Additionally, we found that the cytokine expression increased in the lungs of cigarette smoke-exposed mice compared to air-exposed mice **(** **Supplementary Figure 1D****)**. Although these data suggest differentiation of bone marrow progenitors into hematopoietic cells after smoke exposure, we cannot rule out decreased apoptosis of these cells after smoke exposure. Furthermore, the data suggest a preferential egress of myeloid cells from the bone marrow after smoke exposure.

**Figure 1:**
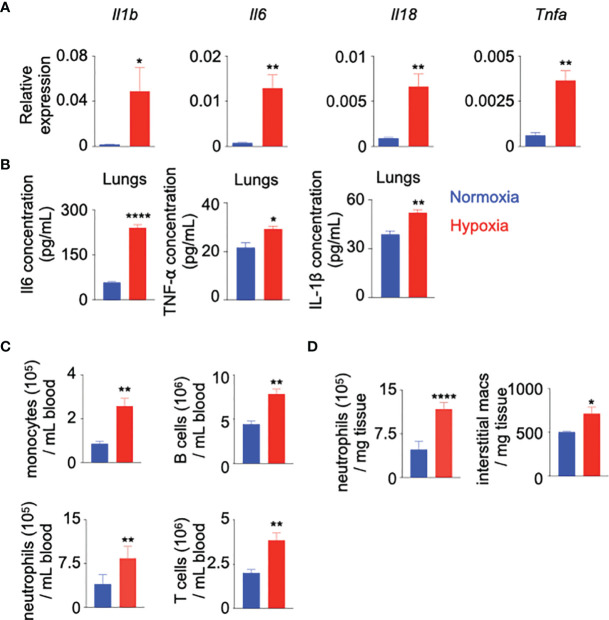
Hypoxia induces inflammation. C57BL/6 mice were placed under either normoxic or hypoxic conditions for 21 days. Lungs, blood and bone marrow were collected. **(A)** *Il1b, Il6, Il18 and Tnfa* expression was assessed by RT-qPCR.**(B)** Il-6, TNF-α and IL1-β protein expression was evaluated in the whole lung and serum by ELISA. **(C)** The numbers of blood monocytes, neutrophils, B and T cells in hypoxic versus normoxic mice were determined by flow cytometry. **(D)** The numbers of lung infiltrating neutrophils and interstitial macrophages in these mice were quantified by flow cytometry. n = 5 mice per condition. Data are shown as mean ± s.e.m. *P < 0.05, **P < 0.01, ****P < 0.001.

To further delineate the contributions of hypoxia in systemic inflammation, we used a mouse model of chronic hypoxia (10% oxygen). We quantified inflammatory cytokines and leukocytes in mice exposed to hypoxic conditions compared to normoxic controls. We observed increased expression of *Il-1b*, *Il-6*, *Il-18* and *Tnfa* in the lungs of hypoxic mice **(** **Figure 1A****)**. Consistent with this heightened expression of the inflammatory cytokine genes, we found elevated concentrations of Il-6, TNF-α and IL-1β in lung parenchyma (**Figure 1B**). Furthermore, we found that mice in chronic hypoxic conditions had increased numbers of monocytes, neutrophils, and B and T lymphocytes in the blood compared to normoxic control mice (**Figure 1C** and **Supplementary Figures 2A, B**). Similarly, the lungs of hypoxic mice harbored augmented numbers of inflammatory cells, including interstitial macrophages (**Figure 1D** and **Supplementary Figure 2C**), which are reported to have pathologic roles in pulmonary diseases characterized by hypoxia (44–46). The frequency of bone marrow monocytes and neutrophils increased after hypoxia exposures (**Supplementary Figures 2C, D**). Additionally, we evaluated leukocyte populations in the spleen of normoxic v. hypoxic mice. We found increased numbers of monocytes and B cells, decreased number of T cells and unchanged numbers of neutrophils **(** **Supplementary Figure 2E****)**. We also measured the ratio of blood/BM leukocyte numbers and noticed a significant increase in this ratio for monocytes and neutrophils suggesting an active egress from the BM to the blood of these cells (**Supplementary Figure 2F**). Altogether, these data demonstrate leukocytosis in patients and mice with hypoxia.

### Hypoxia Increases Proliferation of Hematopoietic Stem and Progenitor Cells

**Figure 2:**
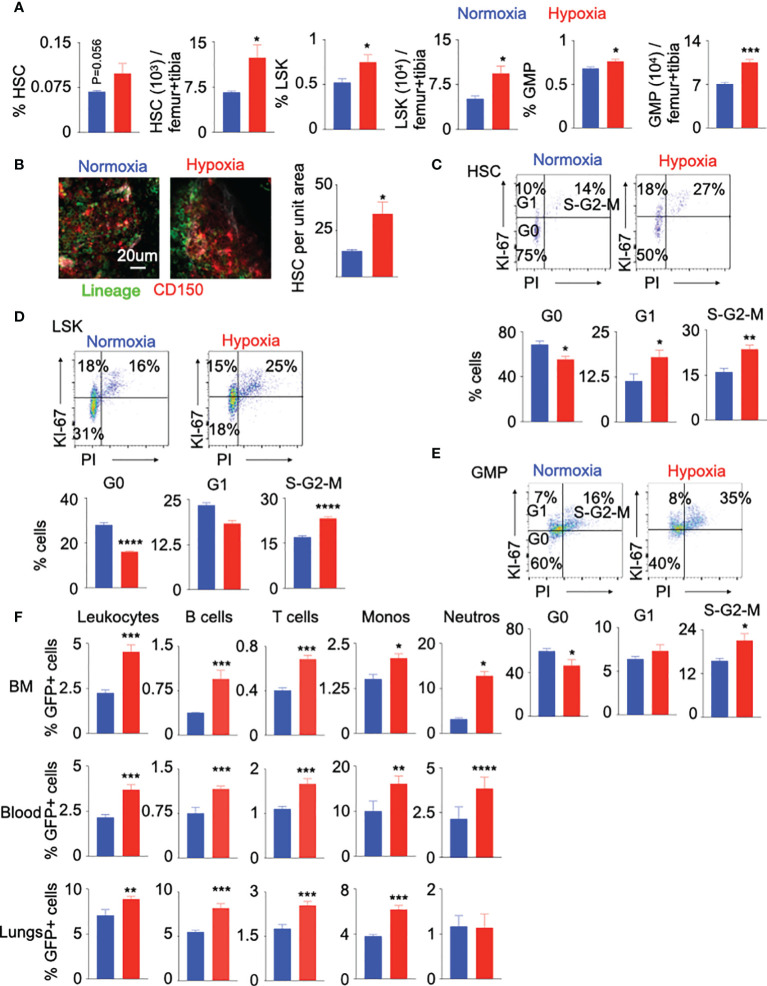
Hypoxia drives HSPC into the cell cycle. C57BL/6 mice were placed under either normoxic or hypoxic conditions for 21 days. Bone marrow was collected. The frequencies and numbers of the hematopoietic progenitors in the bone marrow were determined by flow cytometry **(A)** and whole mount confocal microscopy **(B)**. The frequencies of HSC **(C)**, LSK **(D)** and GMP **(E)** in the cell cycle stages were assessed using intracellular staining of Ki-67 and PI. **(F)** KikGR-GFP^+^ (Kikume Green Red) HSPC were adoptively transferred into mice housed in either normoxic or hypoxic conditions for seven days. GFP^+^ progenies were quantified four days later. n = 5 mice per condition. Data are shown as mean ± s.e.m. *P < 0.05, **P < 0.01, ***P < 0.005, ****P < 0.001.

Increased leukocyte numbers in the blood of hypoxic mice may indicate two underlying mechanisms: A) accelerated egress of bone marrow leukocyte into the blood and B) escalated production of leukocytes by hematopoietic stem and progenitor cells (HSPC) in the bone marrow. To determine whether the observed hypoxia-induced leukocytosis represents increased production of mature leukocytes, we assessed the number and proliferation of bone marrow HSPC, which consist of hematopoietic stem cells (HSC), lineage^-^ Sca-1^+^ c-Kit^+^ (LSK or HSPC) cells and granulocyte macrophage progenitors (GMP) (**Supplementary Figure 3A**). Flow cytometry analysis revealed that hypoxia expanded the numbers of HSC, LSK and GMP in the femur and tibia (**Figure 2A**). Confocal microscopy confirmed a higher density of HSPC in the bone marrow of hypoxic mice compared to the normoxic control (**Figure 2B**). In line with augmented leukocyte and HSPC numbers, mice in hypoxic conditions had higher proportions of proliferating HSC, LSK and GMP (**Figures 2C–E**). Studies have shown that chemotherapy and inflammatory cytokines can induce phenotypic shift in Sca-1 surface expression, which may lead to contamination of the phenotypic HSC gate with non-HSC cell types (47–49). To assess if hypoxia alters Sca-1 expression on long term and short term HSC, HSPC, and GMP in the bone marrow, we measured mean fluorescent intensity (MFI) of Sca-1 in these cells by flow cytometry. We did not observe any statistical difference in the expression of Sca-1 in the progenitors between the two groups (**Supplementary Figure 3B**). Additionally, we isolated HSPC and GMPs from mice kept under either normoxic or hypoxic conditions for three weeks. qPCR for the cell cycle genes revealed that the progenitors expressed higher levels of these genes in hypoxic conditions (**Supplementary Figure 4A**). HSPC proliferation is prerequisite for their differentiation into leukocytes. To understand if HSPC expansion in hypoxic conditions can result in their higher differentiation, we analyzed a whole genome transcriptome data comparing human CD34^+^ HSPC cultured under hypoxic vs. normoxic conditions (50). A pathway analysis revealed that the genes involved in differentiation of stem cell, cell cycle and stimulation of cells were enriched in HSPC in hypoxic conditions (**Supplementary Figures 4B, 5–7**). To discern if hypoxia accelerates HSPC differentiation, we adoptively transferred GFP^+^ HSPC into mice housed under normoxic and hypoxic conditions, and enumerated progenies by flow cytometry. The bone marrow, blood and lungs of hypoxic mice contained augmented percentages of donor-derived differentiated leukocytes, lymphocytes, monocytes and neutrophils (**Figure 2F** and **Supplementary Figure 8A**). This difference of unleashed leukocyte production could not be attributed to higher engraftment of GFP^+^ HSPC under hypoxia (**Supplementary Figure 8B****).** In summary, these data indicate that hypoxia drives HSPC into the cell cycle and increases their differentiation into inflammatory cells.

### VEGFr1 Expression Is Increased in Progenitor Cells Following Hypoxia Exposure

**Figure 3:**
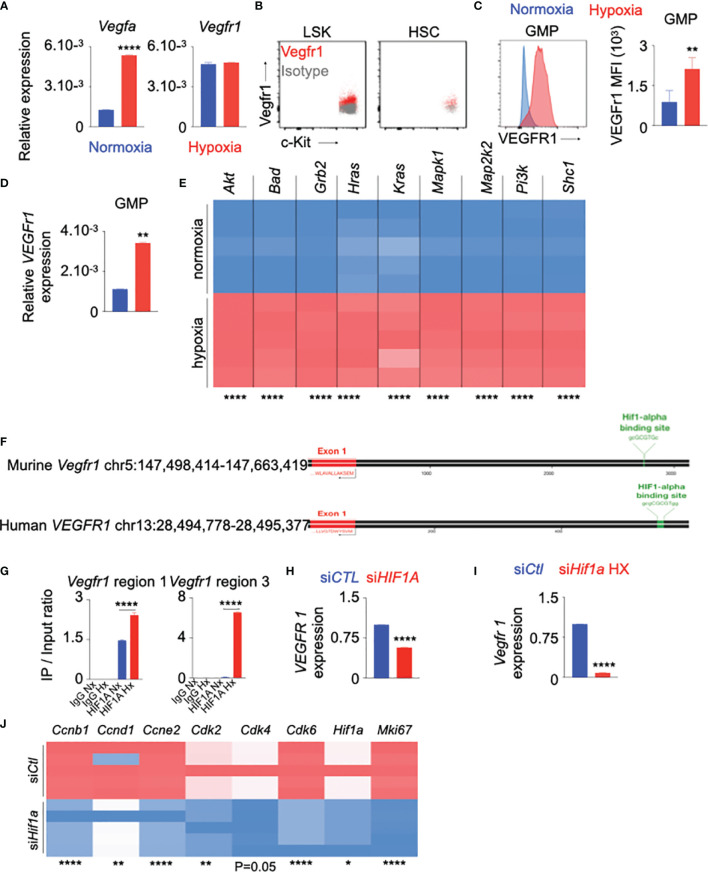
VEGFr1 expression is increased in HSPC in hypoxia. **(A–C)** C57BL/6 mice were placed under either normoxic or hypoxic conditions for 21 days. Bone marrow was collected. **(A)** *Vegfa* and *Vegfr 1* expression in the whole bone marrow was assessed by RT-qPCR. B–C) Vegfr 1 expression was measured in bone marrow LSK and HSC in normoxic conditions **(B)** and GMP in normoxic and hypoxic conditions **(C)** by flow cytometry. The results are represented as mean fluorescent intensity (MFI). **(D)** *Vegfr 1* gene expression was measured in sorted GMP by RT-qPCR. **(E–G)** HSPC were sorted from bone marrow of C57BL/6 mice and cultured in complete SFEM media in either normoxic or hypoxic conditions. **(E)** Heatmap representing expression of the genes downstream to Vegf/Vegfr 1 in HSPC cultured under normoxic and hypoxic conditions. **(F)** Schematic depicting HIF-1α binding sites on both murine and human *VEGFr 1* promoter regions. **(G)** Confirmation of the binding of HIF-1α to the murine *Vegfr 1* promoter region assessed by ChIP qPCR. **(H, I)** *VEGFr 1* expression assessed in human **(H)** and murine **(I)** HSPC transfected with either si*CTL* or si*HIF1A* in hypoxic conditions. **(J)** Heatmap showing the expression of the cell cycle genes in HSPC transfected with either si*Ctl* or si*Hif1a* in hypoxic conditions. n = 5 mice per condition **(A–D)**, 5 replicates per condition **(E–J)**. Data are shown as mean ± s.e.m. *P < 0.05, **P < 0.01, ****P < 0.001.

The role of VEGF/VEGFr1 in hypoxia-induced angiogenesis is well-documented (29, 51, 52). To investigate the possible role of VEGF-A/VEGFr1 in hypoxia-mediated HSPC proliferation, we first assessed RNA expression of these genes in the whole bone marrow of hypoxic mice. The expression of *Vegfa*, but not *Vegfr1*, was increased in bone marrow cells of hypoxic mice compared to their normoxic counterparts (**Figure 3A**). We observed that LSK and HSC express Vegfr1 in the steady state (**Figure 3B**). Additionally, the expression of Vegfr1 mRNA and protein in bone marrow GMP increased in hypoxia (**Figures 3C, D**). In line with these results, we observed that mice exposed to cigarette smoke harbored higher frequency of Vegfr1^+^ HSPC (**Supplementary Figure 9A**). VEGFR1 expression in the progenitors also increased (**Supplementary Figure 9B**). Next, we identified the genes downstream to the VEGFr1 signaling using Ingenuity Pathway Analysis (**Supplementary Figure 9C**). The expression of these genes was elevated in HSPC cultured under hypoxic conditions (**Figure 3E**). These data indicate that hypoxia increases VEGFr1 signaling in HSPC. Hypoxia-inducible factor 1α (HIF-1α), a key transcription factor increased in hypoxia, binds to several genes, and increases their expression (53–56). We observed increased *Hif1a* expression in BM HSPC and GMP of hypoxic mice (**Supplementary Figure 9D**). Flow cytometry revealed increased numbers of Hif-1α^+^ HSPC and GMP in hypoxic mice (**Supplementary Figure 9E**). Additionally, we confirmed decreased expression of prolyl-2 hydroxylase, which accelerates ubiquitination and lysosomal degradation of Hif-1α, in HSPC of hypoxic mice (**Supplementary Figure 9F**). Corresponding to the fact that Hif-1α increases glycolysis, we observed elevated expression of glycolytic genes in bone marrow HSPC of hypoxic mice (**Supplementary Figure 9G**). To decipher the mechanisms of increased Vegfr1 expression in hypoxia, we ascertained if the *VEGFR1* promoter region has HIF-1α binding sites. Our *in silico* analysis revealed that HIF-1α has several binding sites on the *VEGFr1* promoters in both humans and mice (**Figure 3F**). Chromatin immunoprecipitation experiments confirmed that HIF-1α binds to the *Vegfr1* promoter in mouse HSPC (**Figure 3G**). HIF-1α inhibition significantly diminished hypoxia-mediated expression of *VEGFr1* in human (**Figure 3H**) and mouse (**Figure 3I**) HSPC. To delineate the role of HIF-1α in hypoxia-induced HSPC proliferation, we silenced this transcription factor in mouse HSPC and observed significant downregulation of the genes involved in cell cycle progression (**Figure 3J**) as well as the genes downstream to the VEGFr1 signaling (**Supplementary Figure 9H**). In aggregate, these data indicate that HIF-1α directly binds to the *VEGFr1* promoter in HSPC, increases *VEGFr1* expression and augments hematopoietic progenitor proliferation under hypoxic conditions.

### VEGFr Inhibition Decreases HSPC Proliferation

**Figure 4:**
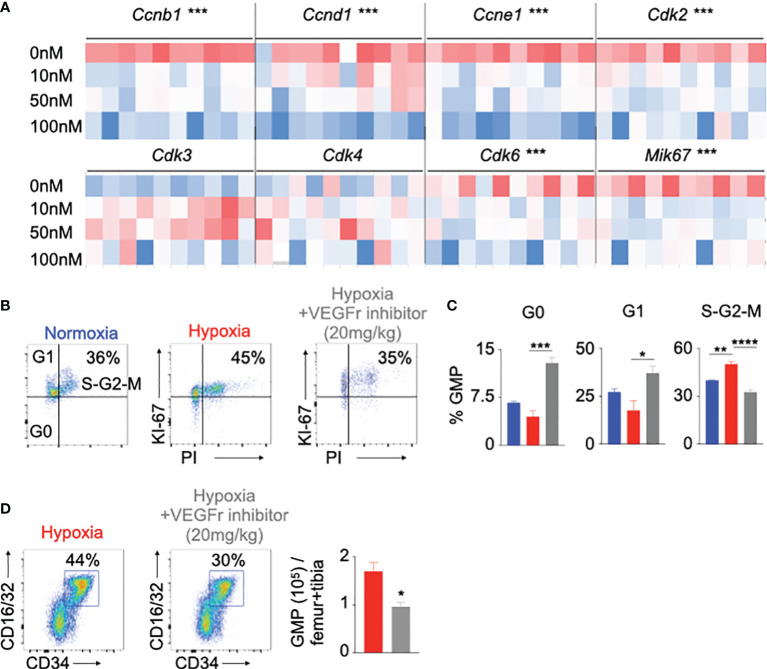
Vegfr inhibition decreased hematopoietic progenitor proliferation in hypoxia. **(A)** Heatmap depicting expression of the cell cycle genes in mouse HSPC treated with increasing amounts of a Vegfr inhibitor (SU5416, 10-100nM) or vehicle. **(B-D)** C57BL/6 mice were treated with either SU5416 or vehicle diluent and placed in either normoxia or hypoxia for 21 days. Bone marrow cells were analyzed using flow cytometry to assess GMP proliferation **(B, C)** and number **(D)**. n = 5 replicates or mice per condition. Data are shown as mean ± s.e.m. *P < 0.05, **P <. 0.01, ***P < 0.001, ****P < 0.001.

The role of the VEGF signaling is well documented in angiogenesis in different diseases such as cancer and age-related macular degeneration (23–25, 52, 57). However, the contribution of VEGFr in HSPC proliferation is not well studied. To understand the role of VEGFr in hypoxia-mediated HSPC proliferation, we cultured HSPC sorted from mouse bone marrow with SU5416, a VEGFr inhibitor (58, 59), under hypoxic and normoxic conditions. VEGFr inhibition significantly decreased the expression of cell cycle genes responsible for HSPC proliferation (**Figure 4A** and **Supplementary Figure 10A**). However, SU5416 inhibits both Vegfr1 and Vegfr2. To ascertain if Vegfrr2 is also important in hypoxia-mediated HSC proliferation, we measured Vegfr2 expression in bone marrow cells of normoxic and hypoxic mice by qPCR. We did not observe any significant difference in Vegfr2 expression in bone marrow cells between these two groups of mice (**Supplementary Figure 10B**). To evaluate the importance of Vegfr2 in HSC proliferation, we have knocked down *Vegfr2* in HSPC isolated from B6 mice using si*Ctl* and si*Vegfr2* and have measured their proliferation. Vegfr2 does not have any significant effect on progenitor proliferation (**Supplementary Figures 10C–F**). To further examine the role of VEGFr signaling in proliferation of HSPC, we assessed proliferation of GMP in hypoxic mice injected with the VEGFr inhibitor. Compared to untreated controls, VEGFr inhibitor-injected mice had lower proportions of GMPs in the S-G2-M cell cycles (**Figures 4B, C**). These mice had increased numbers of quiescent cells in the G0 phase. Interestingly, VEGFr1 inhibition also increased the frequency of GMP in the G1 phase, indicating a G1 to S-G2M arrest in the absence of VEGFr signaling. Additionally, there was a significant reduction in GMP numbers in the bone marrow of hypoxic mice after VEGFr inhibition (**Figure 4D**).

### VEGFr Inhibition Decreased Inflammation and Inflammatory Cell Numbers

**Figure 5:**
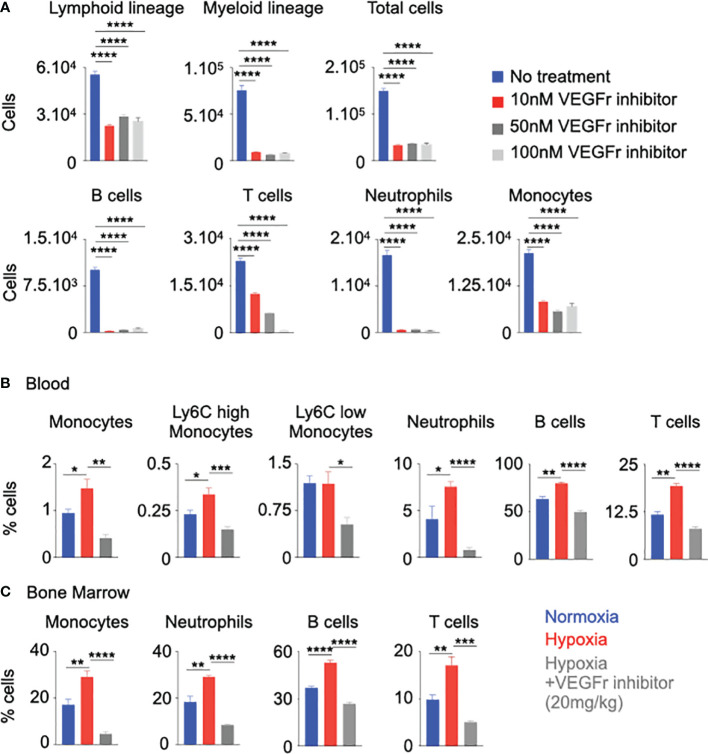
Vegfr inhibition decreased inflammation in hypoxia. **(A)** Mouse HSPC were treated with increasing amounts of a Vegfr inhibitor (SU5416, 10-100nM) or vehicle diluent (no treatment). Lineage commitment of HSPC was assessed by flow cytometry. B&C) C57BL/6 mice were treated with either a Vegfr inhibitor (SU5416) or vehicle diluent and placed in hypoxia for 21 days. Bone marrow and blood leukocytes were analyzed using flow cytometry. Percentages of blood **(B)** and bone marrow **(C)** monocytes and neutrophils were ascertained. n = 5 replicates or mice per condition. Data are shown as mean ± s.e.m. *P < 0.05, **P < 0.01, ***P < 0.001, ****P < 0.0001.

We wanted to evaluate whether the decreased proliferation of HSPC after VEGFr inhibition would have an impact on inflammatory leukocyte generation. To this end, we differentiated HSPC isolated from mouse bone marrow in presence of the VEGFr inhibitor in hypoxic and normoxic conditions. VEGFr inhibition in hypoxic and normoxic HSPC significantly suppressed their differentiation into lymphoid cells, myeloid cells, B cells, monocytes and neutrophils (**Figure 5A** and **Supplementary Figure 11A**). Next, we investigated if VEGFr inhibition decreases hypoxia-induced leukocytosis *in vivo*. Compared to untreated control mice, VEGFr inhibitor-treated mice had decreased percentages and numbers of monocytes and neutrophils in peripheral blood and bone marrow (**Supplementary Figures 11B, C** and **Figures 5B, C**). Additionally, we utilized an additional loss of function approach since SU5416 is not specific for VEGFr1. We silenced *Vegfr1* in sorted murine HSPC placed in hypoxia for 24 hours. We found that the ability of HSPC to differentiate into mature leukocytes, especially B cells, myeloid cells, monocytes and neutrophils, was reduced in HSPC treated with si*Vegfr1* compared to si*Ctl* (**Supplementary Figure 12A**). Additionally, we found decreased expression of the cell cycle check point genes such as *Cdk3, Cdk4*, and *Mki67* after *Vegfr1* knock down (**Supplementary Figure 12B**). Overall, these data indicate the importance of VEGFr1 in the proliferation and differentiation of HSPC into leukocytes under hypoxic conditions.

### Patients With OSA and COPD Recapitulate the Observations Made in Hypoxic Animals

**Figure 6:**
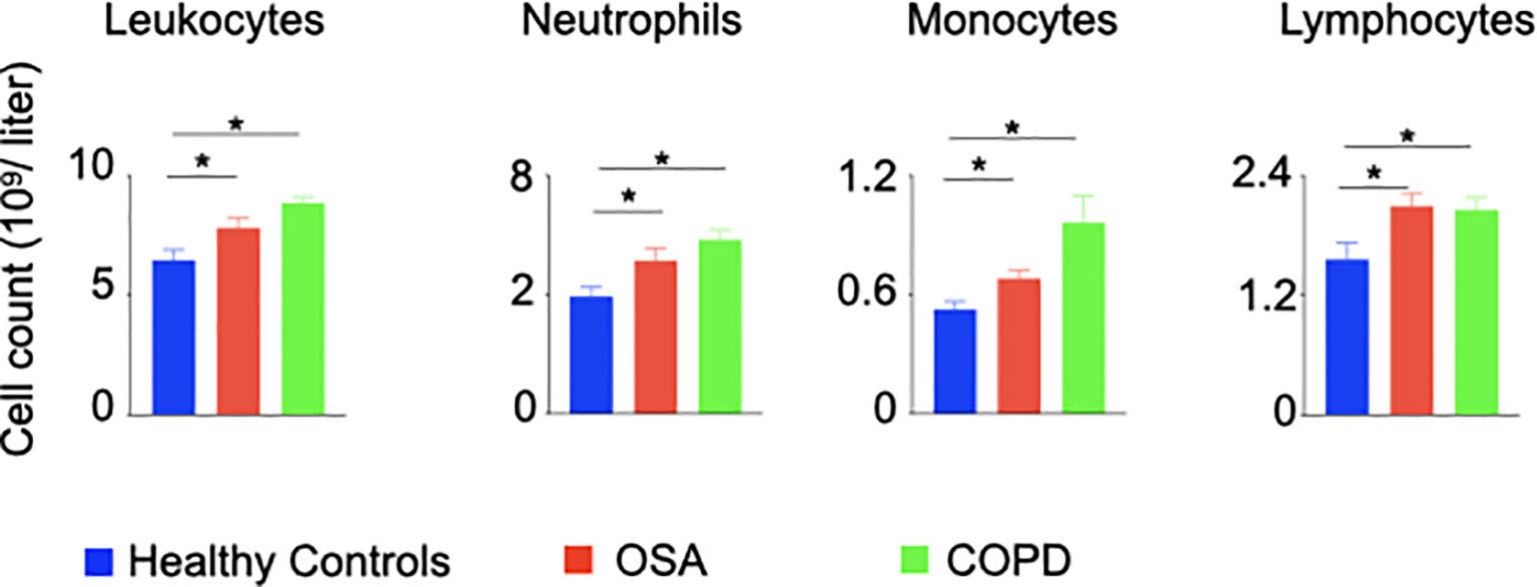
Patients with hypoxia have elevated numbers of inflammatory leukocytes. A restrospective chart review was performed to enumerate inflammatory leukocytes in peripheral blood of patients with OSA and COPD. n = 26 (OSA), 10 (COPD) and 22 (Healthy controls). Data are shown as mean ± s.e.m. *P < 0.05.

To ascertain systemic inflammation in patients with hypoxic lung diseases, we performed a retrospective chart review of patients with OSA characterized by intermittent hypoxia and COPD marked by chronic hypoxia, and age-matched control patients to enumerate inflammatory cells. The total numbers of neutrophils, lymphocytes, and monocytes were significantly increased in the patients with OSA and COPD compared to the healthy controls (**Figure 6** and **Supplementary Figure 13A**). Additionally, the oxygen saturation levels of OSA and COPD patients included in this study were significantly reduced compared to healthy controls (**Supplementary Figure 13B**), indicating hypoxia in these patients.

## Discussion

Patients with hypoxic lung disease, including COPD and OSA, demonstrate chronic inflammation evidenced by elevated inflammatory cytokine levels and inflammatory leukocyte numbers. The degree of systemic inflammation portends clinical outcomes of hypoxic lung disease as well as severity of comorbid conditions (6, 11, 41, 60, 61). There is growing evidence that correction of underlying hypoxia can reduce systemic inflammation highlighting the importance of hypoxia in inflammation (62).

Consistent with serum cytokine levels observed in COPD and OSA, we observed increased levels of IL-1b, IL-6, IL-18, and TNF-a in peripheral blood of mice exposed to hypoxia. Both TNF-a and IL-6 have been shown to be independent risk factors of increased morbidity and mortality in hypoxic lung disease, particularly in COPD (63). IL-6 has been shown to promote proliferation of HSC (64), but interestingly, TNF-α has exhibited a contrasting function regulating hematopoiesis (65). Systemic inflammation has long been postulated to drive the pathogenesis of diseases such as atherosclerosis. The CANTOS trial, assessing monoclonal antibody inhibition of IL-1b, has clearly demonstrated a benefit of attenuating inflammation independent of cholesterol lowering therapies in atherosclerosis (66). Together, these findings illustrate an important role of inflammation in hypoxic lung disease-associated comorbidities.

Elevated numbers of leukocytes in peripheral blood has received recent attention as a marker of inflammation and independent risk factor for diseases such as atherosclerosis (67, 68) and diabetes (69). Lodge et al. illustrated the role of neutrophil-mediated lung damage in patients with COPD (70). Similarly, neutrophilia has been demonstrated as a key feature of obstructive sleep apnea and postulated to contribute to the pathogenesis of OSA (71). The present study demonstrates a mechanism of hypoxia-mediated expansion of inflammatory leukocytes, which likely contribute to the pathogenesis and comorbid conditions of hypoxic lung diseases.

In response to tissue injury, mature leukocytes may be mobilized from sequestered sites or generated *de novo* from hematopoietic tissues (72, 73). It is reasonable to postulate acute increase in recruitment of mature leukocytes from hematopoietic sites contributes to a relative paucity of leukocytes in the bone marrow and abundance in peripheral circulation. In contrast, under chronic inflammation, expansion of HSPC maintains reservoirs of leukocytes in hematopoietic tissues (74, 75). Additionally, studies have shown that HSPC expand profusely in hypoxic conditions *in vitro* (76–78). In line with this, we observed a symmetric expansion of leukocytes in the peripheral blood and bone marrow in hypoxic conditions.

VEGFr1 signaling in the proliferation and differentiation of endothelial cells in angiogenesis, particularly in the development of cancer (79, 80), has been well documented. However, the role of VEGF signaling in the proliferation of hematopoietic stem cells and hematopoiesis is understudied. In line with our findings, a few other studies have shown that inhibition of Vegfr1 diminished HSC cell cycling and lineage differentiation after bone marrow suppression (81), and pharmacological stabilization of HIF-1α increases HSC quiescence (82). Our study demonstrates that the proliferation of HSPC and leukocytosis under hypoxic conditions is mediated by VEGFr1 although the data do not rule out the contribution of VEGFr2 in this process. Thus, the current study identifies VEGFr1 as a novel target to dampen inflammation in diseases characterized by hypoxia. Randomized clinical trials will be required to validate the therapeutic efficacy of this target.

HIF-1α is an important regulator of VEGFA expression in local hypoxia, such as tumor microenvironment (83) and on the development and survival of the hematopoietic system (84, 85). HIF-1α also regulates VEGFA transcription, and mobilization of HSPC increases VEGF-A expression (86). Our in silico and molecular experiments demonstrated that HIF-1α directly interacts with the *VEGFr1* promoters in human and mouse HSPC under hypoxic conditions contributing to increased expression of this receptor. The importance of this finding was further supported by suppression of HSPC proliferation and leukocyte differentiation after *Hif1a* silencing in HSPC. However, we acknowledge that we do not know if higher HSPC differentiation is due to the direct effect of hypoxia/HIF-1α or the resulting inflammation. Although our data show that *Hif1a* silencing decreased HSPC proliferation, HIF-1α also promotes inflammation. Congruently, our and other groups have shown that hypoxia results in systemic inflammation (37, 38, 87). Proinflammatory cytokines can drive HSPC into the cell cycle and increase their proliferation. Thus, exaggerated inflammation may increase lineage output from HSPC in hypoxic mice. Future studies are warranted to understand these mechanisms to reveal potential molecular targets to reduce inflammation in hypoxic diseases.

## Data Availability Statement

Publicly available datasets were analyzed in this study. This data can be found here: https://www.ncbi.nlm.nih.gov/geo/query/acc.cgi?acc=GSE54663.

## Ethics Statement

The studies involving human participants were reviewed and approved by University of Pittsburgh Internal Review Board. The patients/participants provided their written informed consent to participate in this study. The animal study was reviewed and approved by IACUC.

## Author Contributions

JF and SPO conducted experiments, data analysis, and wrote the manuscript. SBV, LLO and AA conducted experiments. SG and SPO helped with the SpO2 measurement experiment. CRK, SG, JCB and JS helped with the experiments involving the mouse model of COPD. SYC reviewed the manuscript and provided us with hypoxic chambers for culturing HSCPC. PD designed the research study and composed the manuscript.

## Conflict of Interest

SYC has served as a consultant for Zogenix, Aerpio, and United Therapeutics. SYC holds research grants from Actelion and Pfizer. SYC has filed patent applications regarding the targeting of metabolism in pulmonary hypertension.

The remaining authors declare that the research was conducted in the absence of any commercial or financial relationships that could be construed as a potential conflict of interest.

## Publisher’s Note

All claims expressed in this article are solely those of the authors and do not necessarily represent those of their affiliated organizations, or those of the publisher, the editors and the reviewers. Any product that may be evaluated in this article, or claim that may be made by its manufacturer, is not guaranteed or endorsed by the publisher.

## Funding

This work was supported by National Institute of Health grants R00HL121076-03, R01HL14396 7, R01HL142629, R01AG069399, AHA Transformational Project Award (19TPA34910142), AHA Innovative Project Award (19IPLOI34760566) and ALA Innovation Project Award (IA- 629694) (to PD), NIH grants R01 HL124021, HL 122596, HL 138437, and UH2/UH3 TR002073 as well as the American Heart Association Established Investigator Award 18EIA33900027 (to SC), and the VMI Postdoctoral Training Program in Translational Research and Entrepreneurship in Pulmonary and Vascular Biology T32 funded by the National, Heart, Lung and Blood Institute (NHLBI) (to JF).
