# Supplementary material for: VEGF Receptor 1 Promotes Hypoxia-Induced Hematopoietic Progenitor Proliferation and Differentiation
Source: Front Immunol. 2022 May 12;13:882484. doi: 10.3389/fimmu.2022.882484 (PMC9133347; doi:10.3389/fimmu.2022.882484)
Supplement: Supplementary file 1 [file Presentation_1.pdf]

Supplemental Figure 1

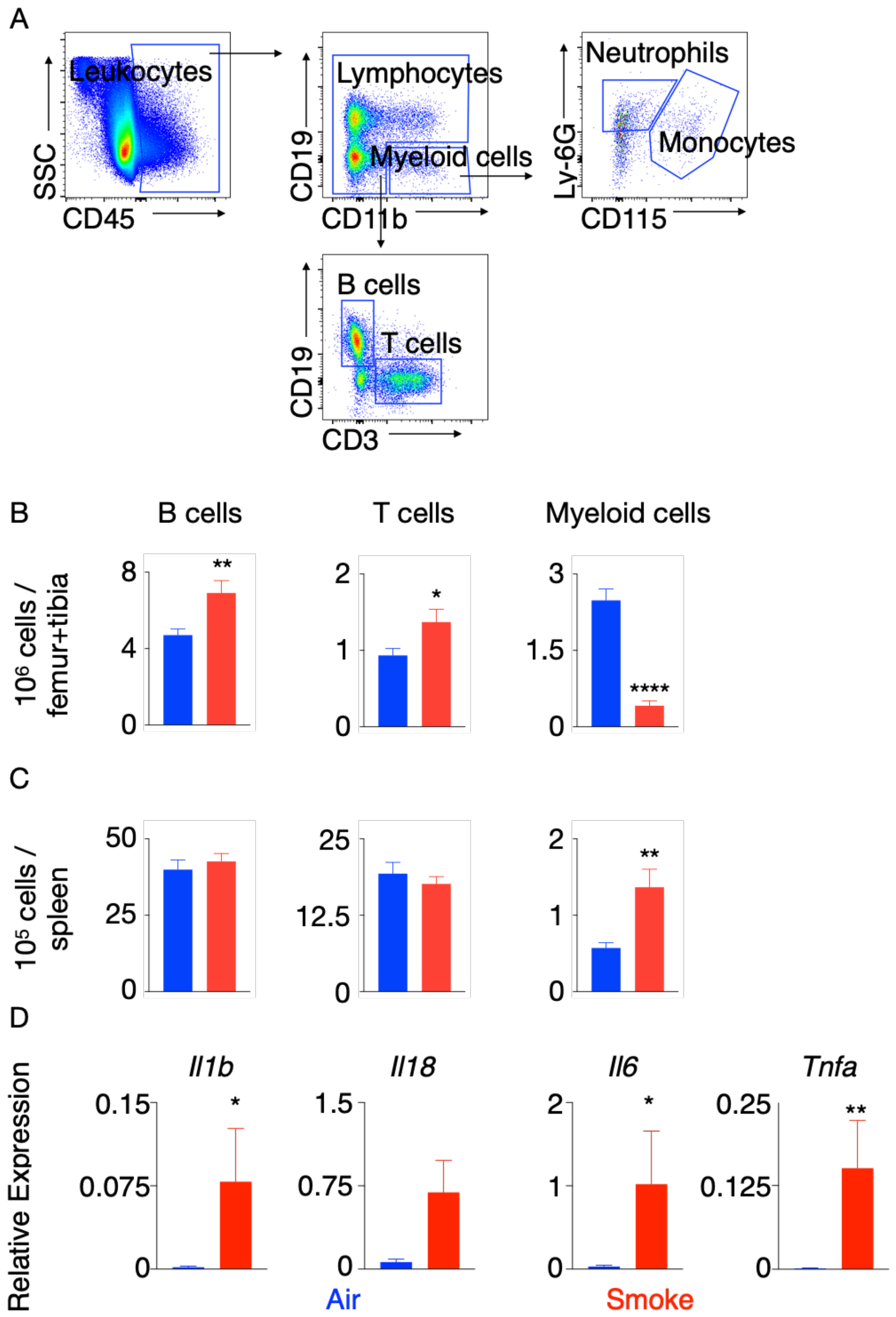

Supplemental Figure 1: Mice exposed to cigarette smoke have altered leukocytes numbers
A. Gating strategy of murine blood leukocytes. B, T and myeloid cells were enumerated in the bone marrow (B) and spleen (C) of these mice. D. mRNA expression of *Il1b, Il18, Il6*, and *Tnfa* in the lungs was measured by RT-qPCR. n=5 samples per condition. Data are shown as mean ± s.e.m. * P < 0.05, ** P < 0.01, **** P<0.001.

Supplemental Figure 2

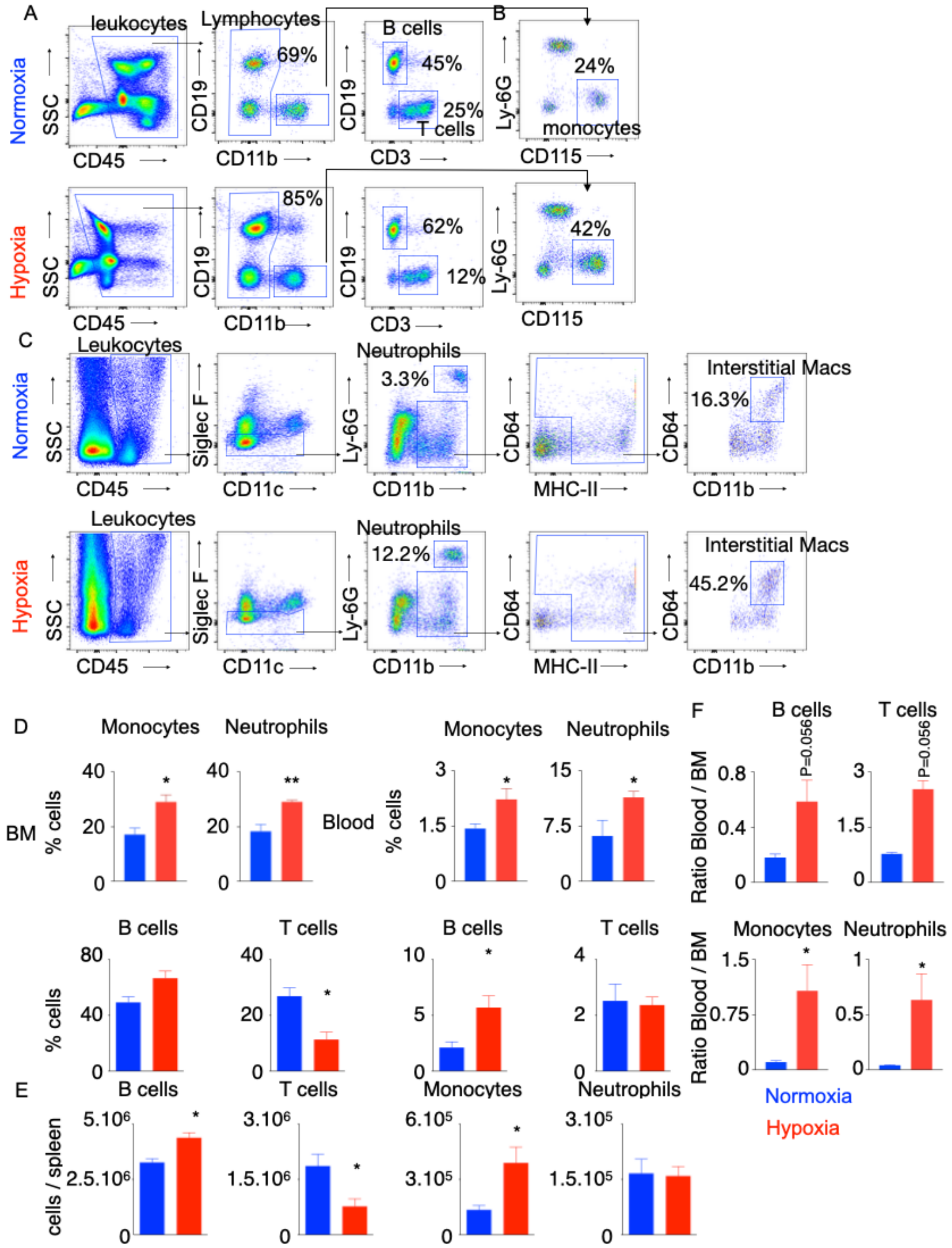

Supplemental Figure 2: The percentages of monocytes and neutrophils are increased in the bone marrow and blood of hypoxic mice.

A-C. Gating strategy of murine blood B and T cells (A), monocytes (B), and lung neutrophils and interstitial macrophages (C). D. The percentages of bone marrow (upper panel) and blood (middle panel) monocytes, neutrophils, B and T cells were evaluated by flow cytometry. E. The numbers of splenic monocytes, neutrophils, B and T cells have been assessed by Flow cytometry. F. The ratios of blood and bone marrow monocytes and neutrophils (lower panel) were calculated in normoxic and hypoxic mice. n=5 mice per condition. Data are shown as mean ± s.e.m. * $P < 0.05$, ** $P<0.01$.

Supplemental Figure 3

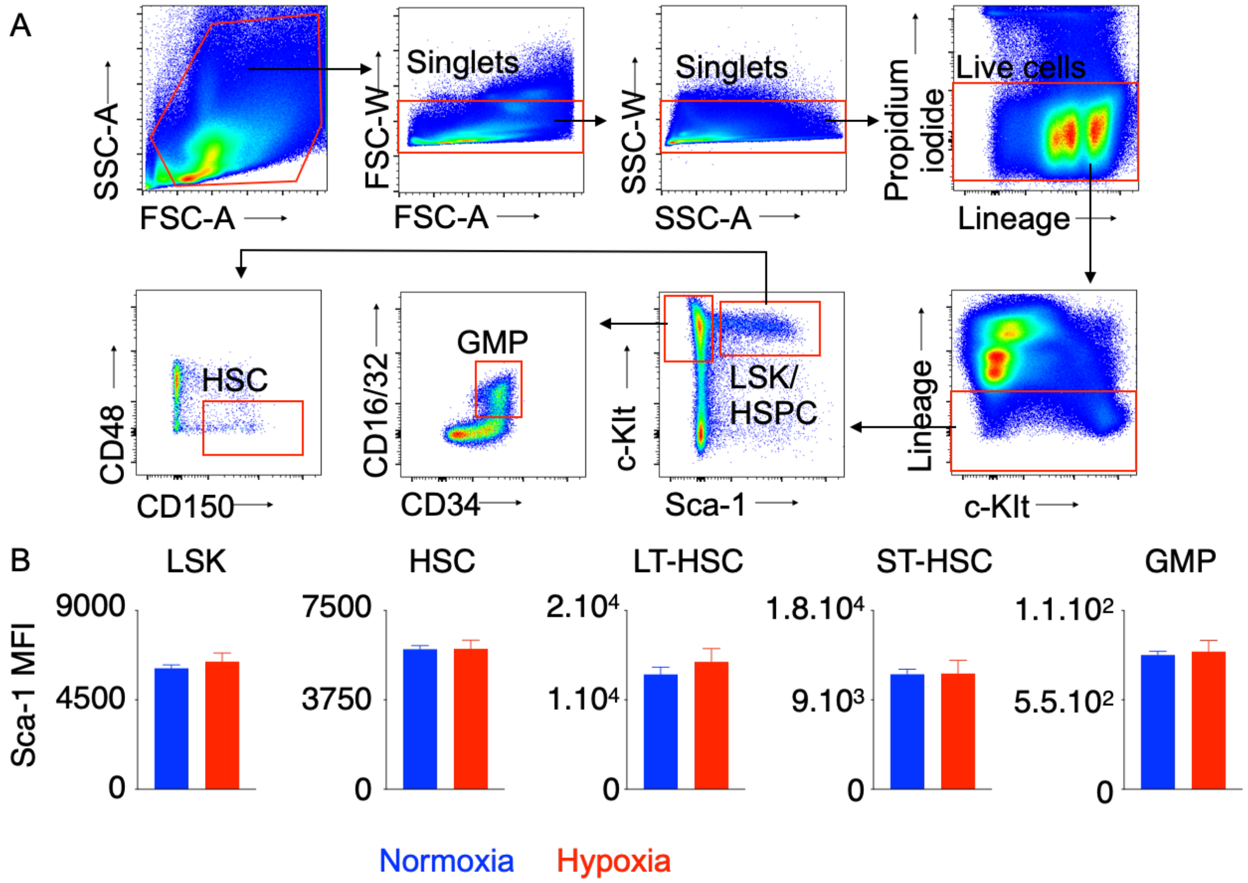

Supplemental Figure 3: Human HSPC flow cytometry gating strategy.

A. Flow cytometric plots showing the gating strategy of murine bone marrow LSK (Lineage$^-$ Sca-1$^+$ c-kit$^+$), HSC (Lineage$^-$ Sca-1$^+$ c-kit$^+$ CD48$^-$ CD150$^+$) and GMP (Lineage$^-$ Sca-1$^-$ c-kit$^+$ CD16/32$^+$ CD34$^+$). B. Mice were subjected to either normoxia or hypoxia for 3 weeks. Sca-1 expression was measured by flow cytometry in bone marrow LSK, HSPC, LT- and ST-HSC as, and GMP. Data are shown as mean +- S.E.M.

Supplemental Figure 4

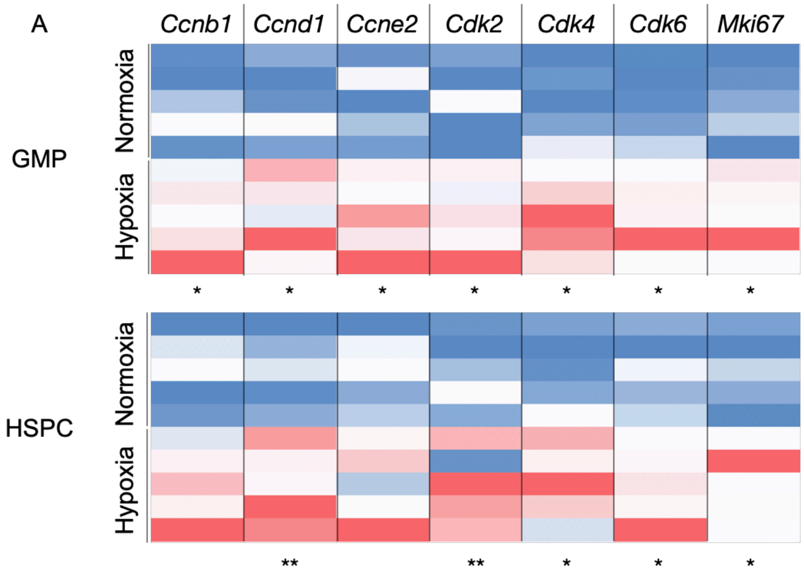

B

ID	Prediction	Expr Log Ratio
PROX1	Increased	7.075
KDR	Affected	6.342
mir-223	Decreased	4.988
WNT5A	Increased	4.974
NTF3	Increased	4.34
APLN	Increased	3.73
DNMT3L	Increased	3.236
CDX1	Increased	3.23
GPR158	Increased	2.906
SFRP2	Increased	2.862
DKK 1	Decreased	2.695
VEGFA	Increased	2.477
WNT11	Increased	2.384
PLXNB2	Increased	2.184
FGFR2	Increased	2.178
BMP4	Increased	2.178
TNFSF11	Increased	2.11
WNT3A	Decreased	2.053
IFNB1	Increased	2.011
NEUROG1	Affected	-2.046
TGFB2	Decreased	-2.193
GATA3	Increased	-2.361
CSF2	Decreased	-2.461
NGF	Decreased	-2.591
GDF6	Increased	-2.591
CSF3	Decreased	-2.82
GPM6A	Affected	-3.257
BHLHE41	Increased	-4.111
GDF7	Decreased	-4.135
MYOC	Decreased	-6.387

Supplemental Figure 4: Hypoxia increases cell cycle gene expression in GMP and HSPC.
A. Heatmap showing the expression of the cell cycle genes in GMP (upper panel) and HSPC (lower panel) sorted from mice exposed to normoxia or hypoxia. B. Analysis of whole genome transcriptome data comparing human CD34$^+$ HSPC cultured under hypoxic vs. normoxic conditions. n = 5 replicates per condition. Data are shown as mean ± s.e.m. * P < 0.05, ** P<0.01.

Supplemental Figure 5

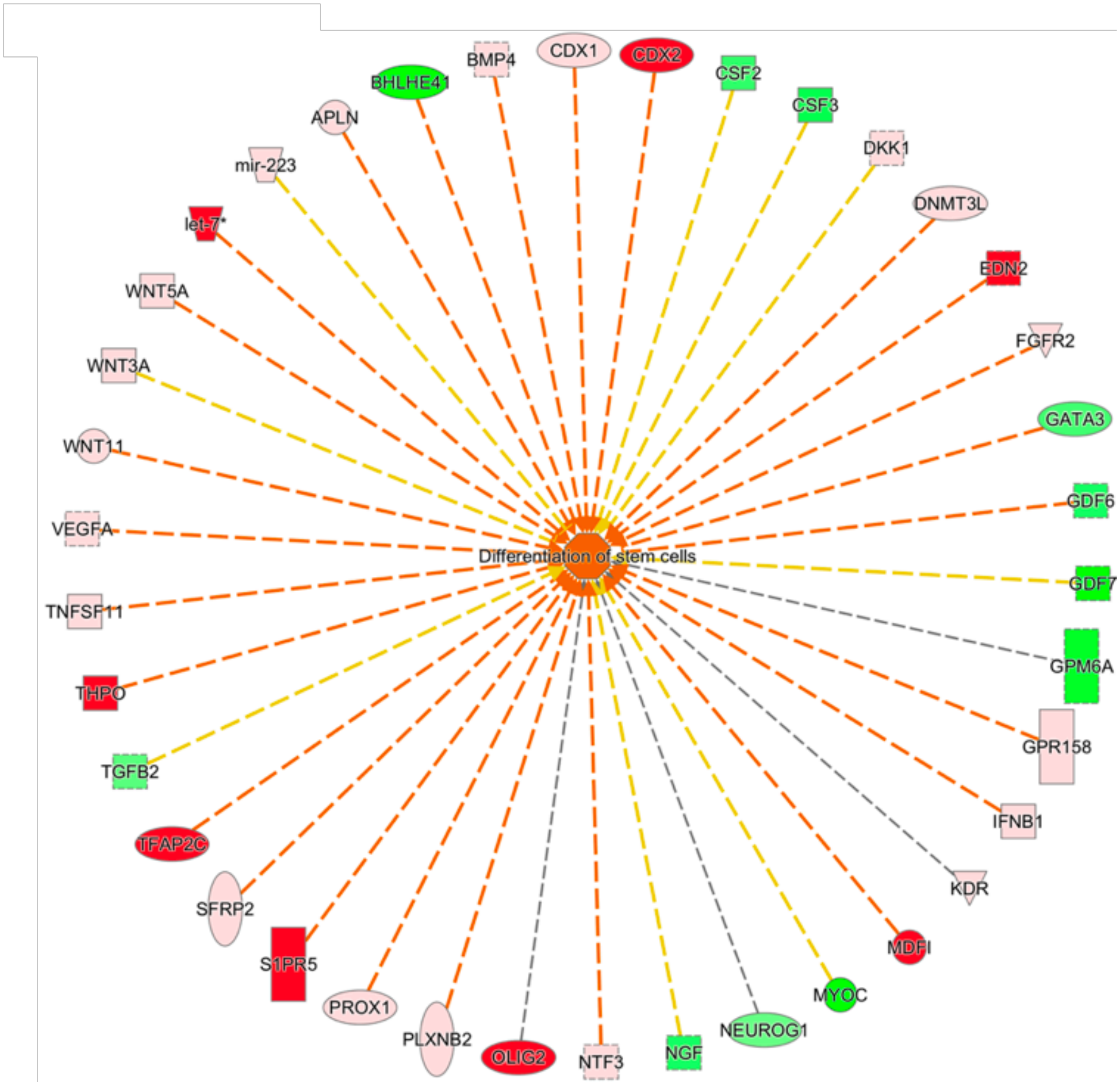

Supplemental Figure 5: Pathway Analysis showing the genes involved in the differentiation of stem cells.

Supplemental Figure 6

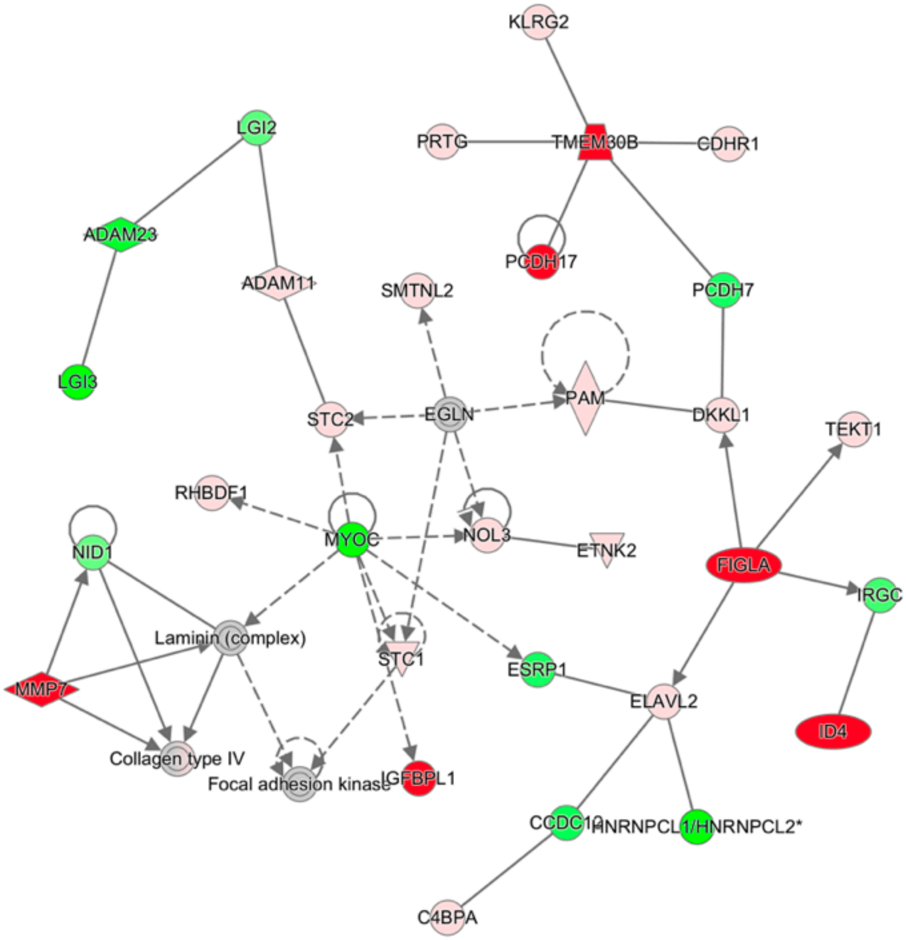

Supplemental Figure 6: Pathway Analysis showing the genes involved in the cell cycle.

Supplemental Figure 7

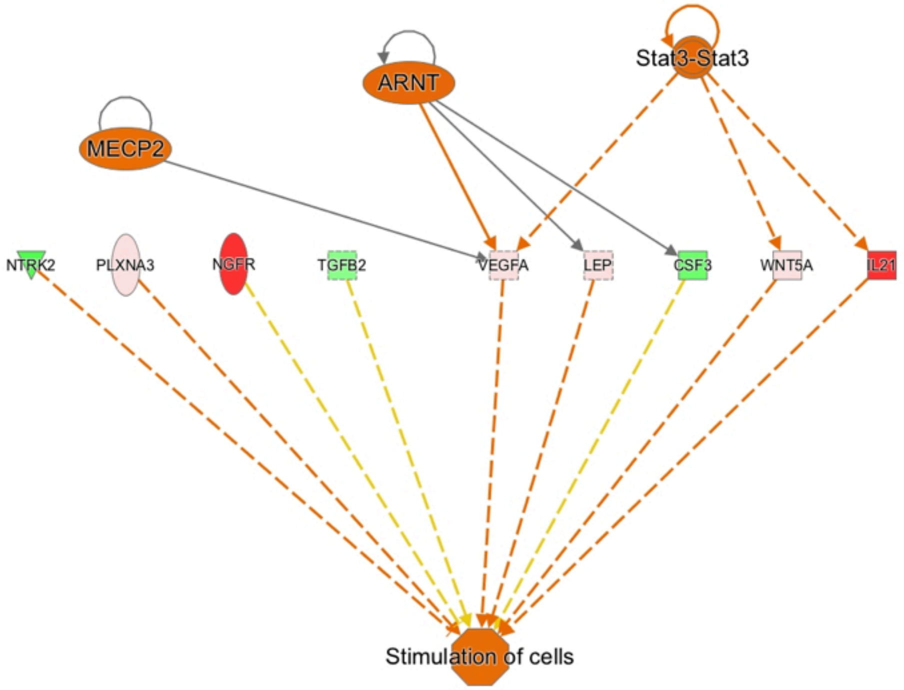

Supplemental Figure 7: Pathway Analysis showing the genes involved in the stimulation of cells.

Supplemental Figure 8

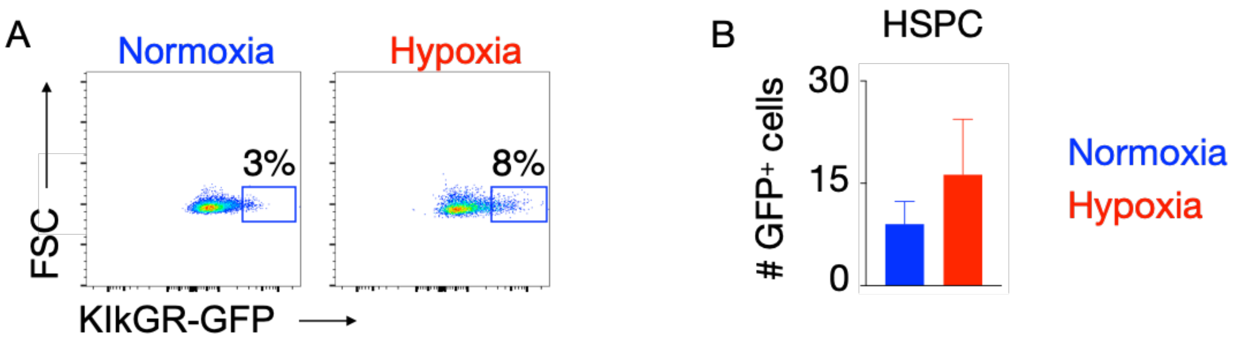

Supplemental Figure 8: The engraftment of GFP$^+$ HSPC was not altered by hypoxic conditions. A. Representative flow cytometric plots showing donor KikGR-GFP$^+$ leukocytes. B. GFP$^+$ HSPC were enumerated in the bone marrow one day after their transfer. n=5. Data are shown as mean ± s.e.m.

Supplemental Figure 9

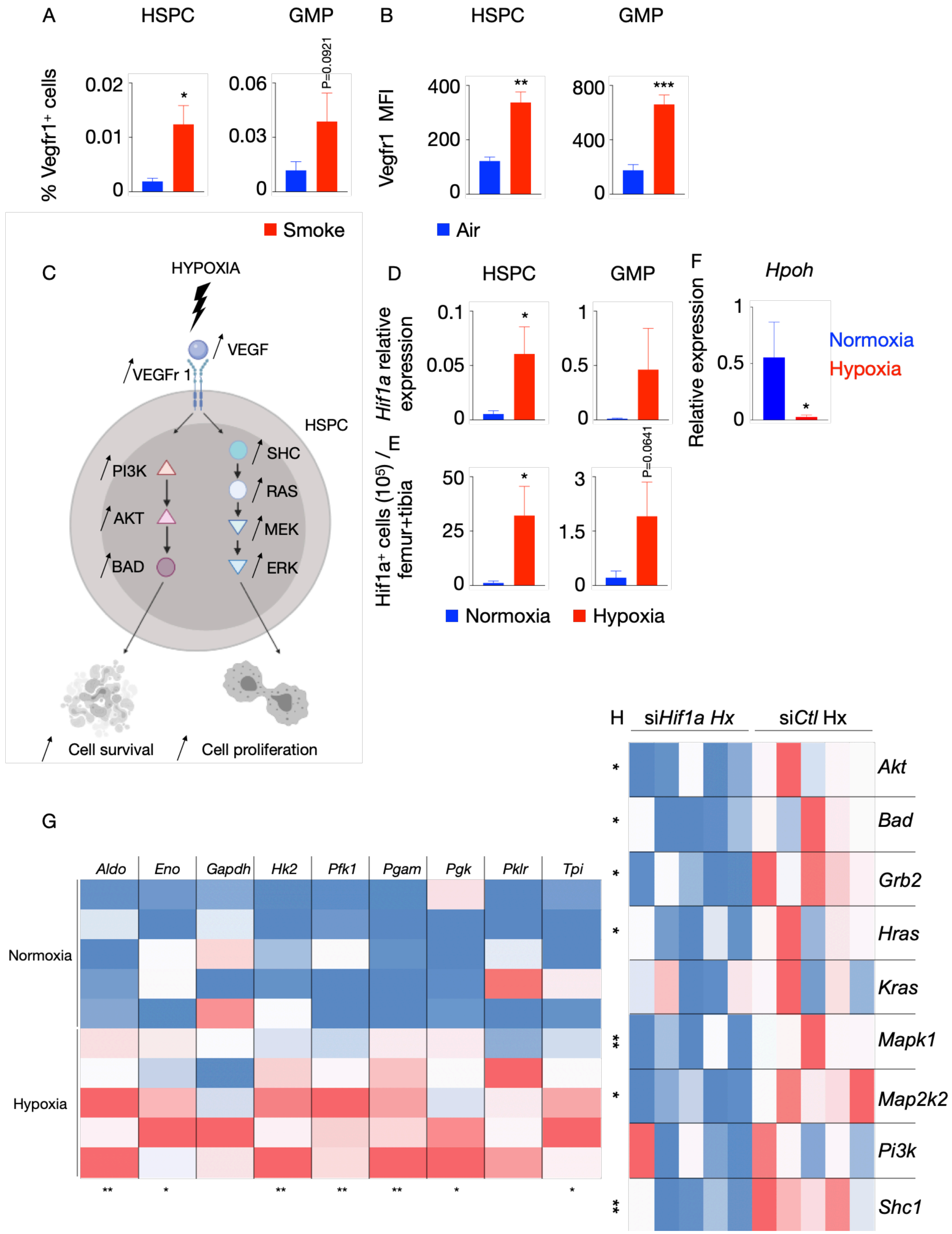

Supplemental Figure 9: The VEGF/VEGFR1 pathways promote cell survival and cell proliferation.

A-B. C57BL/6 mice were exposed to either air (control) or cigarette smoke for six months. A. Bone Marrow Vegfr1^{+} HSPC and GMP were enumerated by flow cytometry. B. Vegfr1 MFI was assessed in bone marrow HSPC and GMP. C. Graphical depiction of the VEGF/VEGFR1 dowstream pathways important for cell survival and proliferation. When VEGF binds to its receptor VEGFR1, this promotes activation of BAD through the mobilization of PI3K/AKT. In parallel, the activation of the MAPK pathway (SHC, RAS, MEK and ERK1/2) promotes cell survival. This cartoon was created using Biorender.com. D-E. Hif-1α expression, and Hif-1α^{+} HSPC and GMP were quantified by qPCR (D) and flow cytometry (E), respectively. F. *Hpoh* expression was assessed in HSPC sorted from normoxic and hypoxic mice by qPCR. G. Heatmap showing the mRNA expression of the glycolytic genes in sorted HSPC of normoxic vs hypoxic mice. H. Heatmap representing expression of the genes downstream to Vegf/Vegfr1 in HSPC treated with either si*Hif1a* or si*Ctl* and cultured under hypoxic conditions. n= 5 samples per condition. Data are shown as mean ± s.e.m. *P < 0.05, **P< 0.01, ***P < 0.005.

Supplemental Figure 10

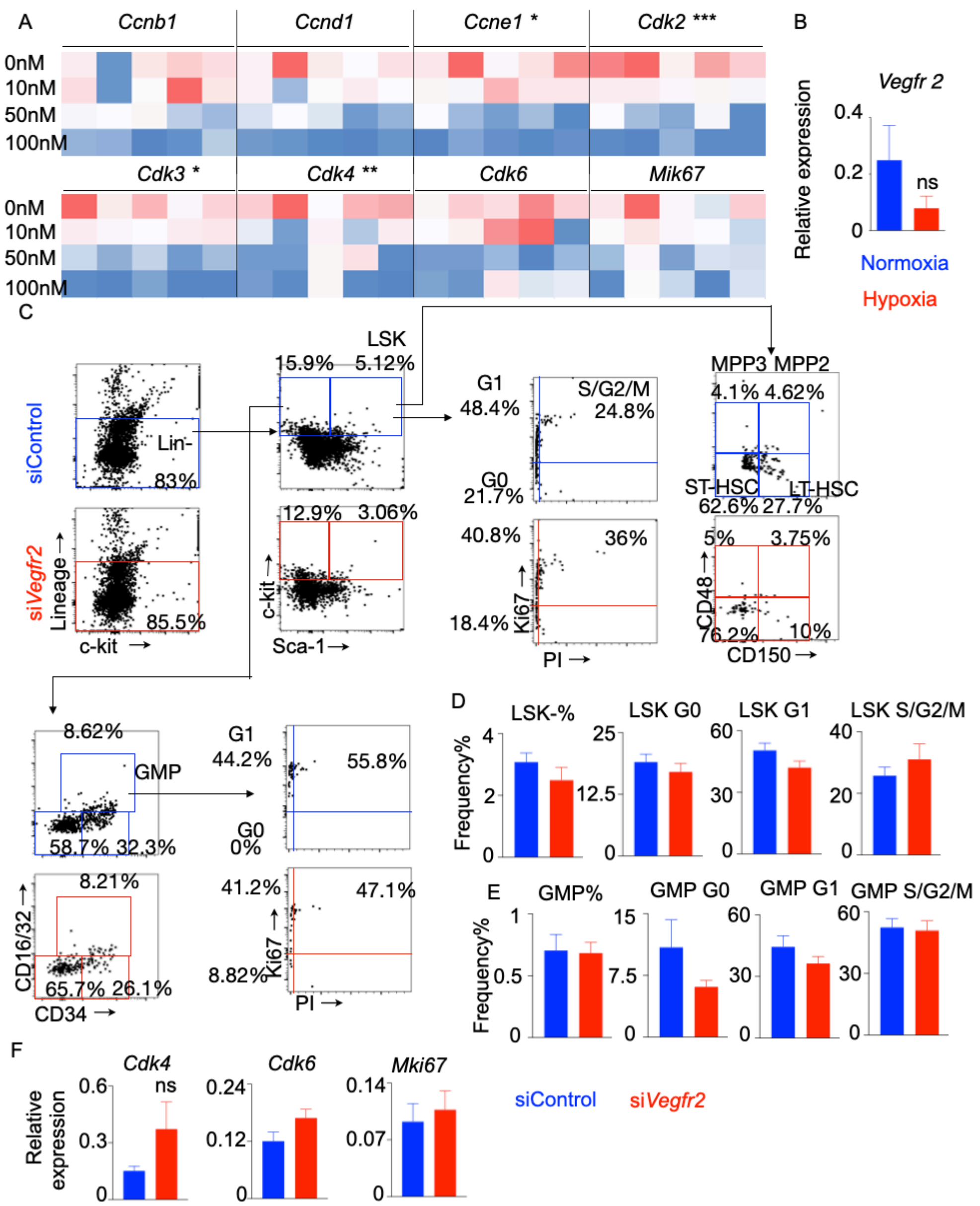

Supplemental Figure 10: *Vegfr* inhibitor treatment decreases HSPC differentiation in normoxia. A. Murine HSPC were sorted, treated with increasing doses of SU5416 and left in hypoxia for 24 hours. Heatmap showing the expression of the cell cycle check point genes in HSPC. n= 5 samples per condition. B. Mice were subjected to either normoxia or hypoxia for 3 weeks. *Vegfr2* expression was measured by RT-qPCR in bone marrow cells of hypoxic and normoxic mice. C-E. HSCs were isolated from the bone marrow of wildtype mice and treated with si*Vegfr2* or si*Ctl* for 5 days. C. The representative flow cytometric plots and the gating strategies have been shown. LSK (D) and GMP (E) proliferation were measured by flow cytometry. (F) qPCR was performed to measure cell cycle gene expression in HSPC. Data are shown as mean ± s.e.m. *P < 0.05, **P< 0.01, *** P<0.005.

Supplemental Figure 11

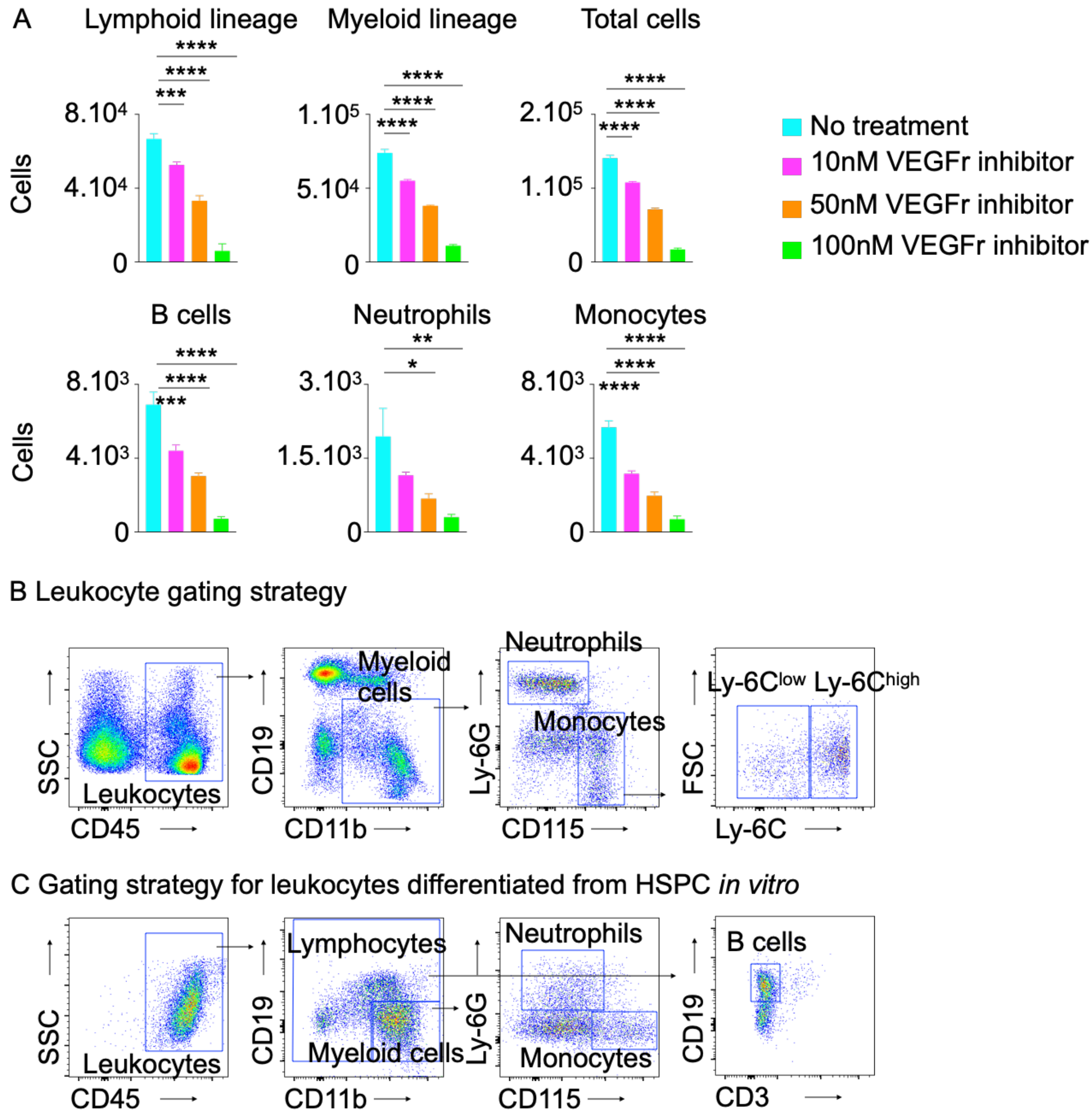

Supplemental Figure 11: *Vegfr* inhibitor treatment decreases HSPC proliferation and differentiation in normoxia.

Murine HSPC were sorted, treated with increasing doses of SU5416 and left in normoxia for 24 hours. A. Bar graphs showing leukocyte enumeration by flow cytometry. B. Flow cytometric plots showing the gating strategy of murine bone marrow and blood neutrophils ($CD45^+$ $CD19^-$ $CD11b^+$ $Ly\text{-}6g^+$ $CD115^-$) and monocytes ($CD45^+$ $CD19^-$ $CD11b^+$ $Ly\text{-}6g^-$ $CD115^+$ $Ly\text{-}6c^{low}$ or $Ly\text{-}6c^{high}$). C) Flow cytometric plots showing the gating strategy for the enumeration of leukocytes differentiated from HSPC *in vitro*. Leukocyte ($CD45^+$), lymphocyte ($CD45^+$ $CD11b^-$), monocyte ($CD45^+$ $CD11b^+$ $CD115^+$ $Ly\text{-}6c^{low}$ or ^{high}) and neutrophil ($CD45^+$ $CD11b^+$ $Ly\text{-}6g^+$) gates have been shown. n= 5 samples per condition. Data are shown as mean ± s.e.m. *$P < 0.05$, **$P < 0.01$, ***$P<0.005$, ****$P<0.001$.

Supplemental Figure 12

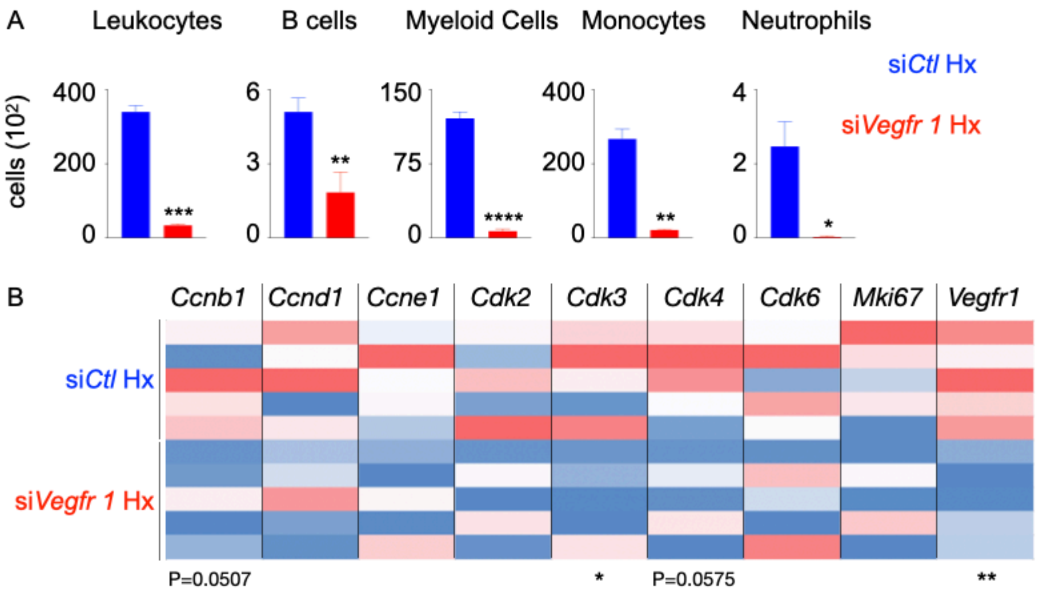

Supplemental Figure 12: *Vegfr1* silencing decreases HSPC differentiation and proliferation in hypoxia. Murine HSPC were sorted, treated with si*Vegfr1* and placed in hypoxia for 24 hours. A. Bar graphs showing leukocyte enumeration by flow cytometry. B. Heatmap showing the expression of the cell cycle check point genes in HSPC. n= 5 samples per condition. Data are shown as mean ± s.e.m. *P < 0.05, **P< 0.01, ***P<0.005, ****P<0.001.

Supplemental Figure 13

A

	Age	Gender	BMI	Ethnicity
HC1	43	F	37.55	AA
HC2	27	F		NHW
HC3	87	F	20.68	NHW
HC4	57	F	39.04	NHW
HC5	26	F	25.64	AA
HC6	63	F	36.4	NHW
HC7	28	F	25.61	AI
HC8	42	F	26.36	NHW
HC9	66	F	41.8	NHW
HC10	68	F	35.03	AA
HC11	57	F	29.7	H
HC12	50	F	29.24	AA
HC13	56	F	52.03	AA
HC14	53	F	25.78	AA
HC15	31	F	21.08	NHW
HC16	28	F	36.73	NHW
HC17	54	F	38.75	NHW
HC18	79	M	22.91	NHW
HC19	88	M	23.23	NHW
HC20	37	M	28.76	S
HC21	63	M	36.7	NHW
HC22	57	M	30.94	NHW
AP1	55	F	36.88	NHW
AP2	30	F	25.03	NHW
AP3	64	F	22	NHW
AP4	72	F	34.05	AA
AP5	38	F	23.43	NHW
AP6	72	F	41.69	NHW
AP7	50	F	52.45	AA
AP8	76	F	43.67	NHW
AP9	61	F	34.78	NHW
AP10	70	F	36.95	NHW
AP11	55	F	45.93	NHW
AP12	58	F	40.69	NHW
AP13	81	M	26.6	NHW
AP14	58	M	37.7	NHW
AP15	67	M	36.2	Not Specified
AP16	52	M	31.82	NHW
AP17	54	M	40.39	AA
AP19	84	M	25.92	NHW
AP20	42	M	34.79	NHW
AP21	55	M	32.93	NHW
AP22	57	M	40.7	AA
AP23	79	M	26.02	NHW
AP24	65	M	26.37	NHW
AP25	81	M	28.81	NHW
AP26	65	M	28.93	NHW
COPD1	63	M	34.98	NHW
COPD2	59	F	19.16	NHW
COPD3	60	F	43.2	NHW
COPD4	77	F	32.34	NHW
COPD5	85	F	29.6	NHW
COPD6	84	M	27.73	AA
COPD7	57	M	34.11	NHW
COPD8	70	M	31.27	NHW
COPD9	67	F	28	NHW
COPD10	59	M	39.61	NHW

B Oxygen Saturation

	Healthy Controls	OSA	COPD
	96	90	90
	97	95	91
	95	93	90
	95	90	85
	95	92	92
	95	90	77
	97	90	88
	97	85	93
	89	88	84
	90	90	88
	95	91	
	88	79	
	89	92	
	93	88	
	95	88	
	92	87	
	94	87	
	91	89	
	93	94	
	94	93	
	96	92	
	93	93	
		93	
		89	
		87	
Average	93.59090909	89.8	87.8

AA = African American
NHW = Non-hispanic white
AI = American Indian
H = Hispanic
S = Asian

Supplemental Figure 13: List of human patients and healthy controls

A. Table presenting a retrospective chart review of patients with OSA characterized by intermittent hypoxia (n=26, age average=61.64±13.71) and COPD marked by chronic hypoxia (n=10, age average=68.10±10.56), and age-matched control patients (n=22, age average=52.73±18.69) to enumerate inflammatory cells. B. Table displaying the oxygen saturation of healthy controls, and patients with OSA and COPD.
